# Boosting Face Presentation Attack Detection in Multi-Spectral Videos Through Score Fusion of Wavelet Partition Images

**DOI:** 10.3389/fdata.2022.836749

**Published:** 2022-07-22

**Authors:** Akshay Agarwal, Richa Singh, Mayank Vatsa, Afzel Noore

**Affiliations:** ^1^Department of Computer Science and Engineering, Indraprastha Institute of Information Technology Delhi (IIIT-Delhi), New Delhi, India; ^2^Department of Computer Science and Engineering, Indian Institute of Technology (IIT) Jodhpur, Jodhpur, India; ^3^Department of Computer Science and Electrical Engineering, Texas A&M University, KIngsville, TX, United States

**Keywords:** face recognition (FR), presentation attack detection (PAD), multi-spectral, security, generalized PAD

## Abstract

Presentation attack detection (PAD) algorithms have become an integral requirement for the secure usage of face recognition systems. As face recognition algorithms and applications increase from constrained to unconstrained environments and in multispectral scenarios, presentation attack detection algorithms must also increase their scope and effectiveness. It is important to realize that the PAD algorithms are not only effective for one environment or condition but rather be generalizable to a multitude of variabilities that are presented to a face recognition algorithm. With this motivation, as the first contribution, the article presents a unified PAD algorithm for different kinds of attacks such as printed photos, a replay of video, 3D masks, silicone masks, and wax faces. The proposed algorithm utilizes a combination of wavelet decomposed raw input images from sensor and face region data to detect whether the input image is bonafide or attacked. The second contribution of the article is the collection of a large presentation attack database in the NIR spectrum, containing images from individuals of two ethnicities. The database contains 500 print attack videos which comprise approximately 1,00,000 frames collectively in the NIR spectrum. Extensive evaluation of the algorithm on NIR images as well as visible spectrum images obtained from existing benchmark databases shows that the proposed algorithm yields state-of-the-art results and surpassed several complex and state-of-the-art algorithms. For instance, on benchmark datasets, namely CASIA-FASD, Replay-Attack, and MSU-MFSD, the proposed algorithm achieves a maximum error of 0.92% which is significantly lower than state-of-the-art attack detection algorithms.

## 1. Introduction

Face recognition algorithms have been gaining more interest than ever, both for their increasing usage (Guo and Zhang, 2019; Sepas-Moghaddam et al., 2019) and their limitations (Agarwal et al., 2016, 2019c; Siddiqui et al., 2016; Cavazos et al., 2019; Marcel et al., 2019; Mukudi and Hills, 2019; Wu et al., 2019; Ghosh et al., 2020; Singh et al., 2020). While researchers are attempting to make the face recognition algorithms generalizable to unseen scenarios, it is important that the face presentation attack detection (PAD) algorithms are also generalizable and inclusive (Agarwal et al., 2019a,b; Sanghvi et al., 2021). Existing research in face presentation attack detection has primarily focused on detecting different kinds of attacks captured in the visible spectrum. While the algorithms have received nearly perfect classification performance on individual attacks, the focus is on designing unified algorithms for different kinds of presentation attacks in visible images (Liu et al., 2019b; Mehta et al., 2019).

The usage of face recognition algorithms in the near infrared spectrum is also increasing. With surveillance cameras operating in both visible (VIS) and near infrared (NIR) images, the PAD algorithms must be developed for NIR spectrum face images as well. It is well observed that the illustration of attacks in both spectra is quite different, and hence, attack detection in images of different spectrums require specialized algorithms. Realizing this, recently researchers have started working toward PAD in NIR spectrum images as well (Agarwal et al., 2017; Zhang et al., 2020). This research focuses on extending the usability and efficiency of presentation attack detection algorithms in multiple spectrums and ethnicity variations.

### 1.1. Related Study

In this section, we present a brief overview of the existing algorithms in multi-spectrum (visible + near infrared) presentation attack detection. Face presentation attack detection in other than the visible spectrum is still in nascent stages. Pavlidis and Symosek (Pavlidis and Symosek, 2000) presented an algorithm to detect disguised faces system using images captured in the NIR spectrum. Yi et al. (2014) performed experiments with a print attack by printing the photo of 100 clients on coarse paper in both VIS and NIR spectrum. However, the database was not made publicly available. Later, Chingovska et al. (2016) and Raghavendra et al. (2017) also analyzed the vulnerability of face recognition systems in the NIR spectrum. They demonstrated that face recognition systems working in the NIR spectrum are also susceptible to presentation attacks such as replay and print. Therefore, effective presentation attack detection algorithms are required to protect the surveillance system operating in the NIR spectrum. Chingovska et al. (2016) also released the first publicly available VIS and NIR presentation attack database (MSSPOOF). The database comprises images from 21 subjects. Three photos of each client in the VIS and NIR spectrum are selected and printed using the black and white printer. The database contains 70 real access images and 144 attack images for each client. They demonstrated more than 88% Spoof False Accept Rate (SFAR) using the NIR attack on the NIR face recognition system. However, no counter-presentation attack algorithm is presented in this article.

Later, Raghavendra et al. (2016) proposed a presentation attack detection algorithm on the MSSPOOF (multispectral spoof) database using the combination of Laplacian pyramid and Fourier transform. They reported an Average Classification Error Rate (ACER) of 2.1% and 0.74% on the VIS and NIR spectrum, respectively. Raghavendra et al. (2017) prepared the extended multispectral presentation attack database (EMSPAD) from 50 subjects and attack samples were prepared by printing the images from two different printers. The database is captured in seven different spectral bands ranging from 425 to 930 nm. In total, the database contains 7, 000 print attack images and 3, 500 real access images from 50 subjects. A vulnerability of the face recognition system using printed attack samples is shown with perfect SFAR in 680 nm spectra and more than 98% SFAR in 930 nm spectra. Agarwal et al. (2017) have proposed the first large-scale multi-spectrum face presentation attack database consisting of both 2D and 3D attack instruments. The real and attack videos are captured in several constrained and unconstrained environments and in three imaging spectrums namely visible (VIS), near infrared (NIR), and thermal. Liu and Kumar (2018) proposed different CNN architectures to detect the presentation attack using multi-spectral face images. They prepared the presentation attack database from 13 masked subjects and 9 real subjects. The limitation of the study is the unavailability of the database. Sun et al. (2018) proposed consistent measure based presentation attack detection in multi-spectral imaging. Bhattacharjee et al. (2018) have shown the vulnerability of VIS, NIR, and Thermal face recognition using deep CNN models under custom silicone mask attacks. George et al. (2019) presented a new database along with multi-channel CNN for face PAD.

Heusch et al. (2020) have presented a database in multiple spectrums to effectively present a study on the effect of SWIR imaging on presentation attack detection. Zhang et al. (2020) have a multi-spectral database comprising 2D print attacks. Along with that the squeeze and excitation network using ResNet-18 as a backbone network is also proposed to counter presentation attacks. Li et al. (2020) extended the database by incorporating multiple attacks including a 3D print and silica gel mask. The other popular face presentation attack databases are SiW (Liu et al., 2018), SiW-M (Liu et al., 2019b), and OULU-NPU (Boulkenafet et al., 2017b); however, all are captured in the visible spectrum. The details of the existing face presentation studies can also be found in the comprehensive evaluation (Jia et al., 2020b) and handbook (Bhattacharjee et al., 2019; Marcel et al., 2019).

### 1.2. Research Contributions

The literature review shows that there are a few publicly available databases in the NIR spectrum and a significant lack of research on automated algorithms for presentation attack detection in the NIR spectrum images and algorithms that can detect PAD in multiple spectrums. Inspired by these, there are two primary contributions of this research. The first contribution is that we have prepared a large video-based attack database in the NIR spectrum from more than 340 subjects of two different ethnicities: Indian and Chinese. The second contribution of this article is a state-of-the-art face presentation attack detection algorithm for different kinds of attacks in the NIR + VIS spectrum. The efficacy of the proposed algorithm is demonstrated through extensive experiments. The proposed algorithm not only yields effective performance with spectrum variations but is also generalized against attack instruments, ethnicities, and databases. Experimental evaluation of multiple databases shows that the proposed algorithm surpasses several deep learning, non-deep learning, and state-of-the-art algorithms by a significant margin.

The article is organized as follows. In the next section, the proposed large-scale face presentation attack dataset is described along with the experimental protocol used to perform the face PAD task. The section also describes the impact of the proposed dataset in evaluating the sensitivity of the face recognition algorithms. Section 3 describes the proposed unified face PAD algorithm effective in handling not only individual spectrums such as VIS and NIR but also a combination of them. In Section 4, the effectiveness of the proposed algorithm to handle multiple spectrums is evaluated using several existing multi-spectrum datasets including the proposed dataset. Furthermore, to benchmark, our study is similar to existing studies, in Section 5 extensive experimental evaluation has been performed on the standard visible (VIS) spectrum datasets. The extensive comparison on each dataset with state-of-the-art algorithms are presented and showcase that the proposed algorithm outperforms them with a significant margin. Finally, the conclusion and future research directions are briefly described.

## 2. Proposed Spoof-in-NIR Database

As discussed in the previous section, there are few databases in the NIR spectrum. Therefore, to promote the research in this emerging field, we first prepared a large video PAD database in the NIR spectrum, termed the Spoof-in-NIR database. In this section, we present the details of the proposed NIR presentation attack database. The NIR database contains face images from two completely different ethnicities: Indian and Chinese. The database contains the attack videos captured using the printed photograph of 400 genuine users. The database will be made publicly available to the research community^1^.

### 2.1. Camera Setup

To collect the database in the NIR spectrum, a camera is mounted on the tripod, and subjects are asked to stand at a distance of approximately 1 m and look into the camera. To ensure that the videos are captured in a relatively uncontrolled scenario, no other special instructions are given to the subjects. The database is captured in two different environments and background conditions: one inside and the other outside the building at night time. To ensure that the videos are only captured in the NIR spectrum, a visible cut filter is placed in front of the camera. GO-5000-USB camera^2^ is used to capture the videos at the frame resolution of 2, 048 × 2, 560. Frames are captured at the rate of 20 fps and stored as raw pixels so that the quality of images is not degraded because of compression.

### 2.2. Indian Face Presentation Attack Database

Each ethnicity subset comprises two sets: bonafide and attacked. The bonafide/real videos of the Indian database are captured from 152 subjects at night time using a NIR camera. To provide variability in the data, videos are captured at two different locations and comprise variations in background and illumination conditions. The subjects also perform natural motions such as eye blinking and head movement.

To capture the attacked videos, frontal images of all the subjects are first captured using a DSLR camera in day time constrained environment. A black and white printout of frontal images of 150 subjects is taken using an HP color printer and videos of these images are captured to prepare the attacked video subset. Attack videos of the Indian subset are captured in two different sessions with illumination variations. Attack and real videos are captured for a duration of 12 s to exhibit a real world surveillance scenario.

**Figure 1:**
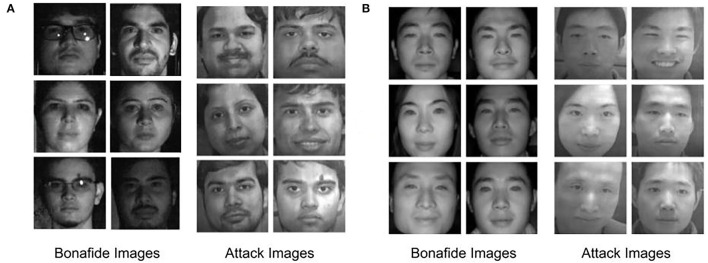
Sample face images from the proposed Spoof-in-NIR database. Images are shown from **(A)** Indian ethnicity and **(B)** Chinese ethnicity.

In total, 300 attacks and 152 real videos are collected as part of this Indian database. The resolution of the real and attack frame is 2, 048 × 2, 560. Face detection is performed using the Viola-Jones face detector (Viola and Jones, 2004). Characteristics of the database are given in Table 1 and samples are shown in Figure 1A.

**Table 1:** Characteristics of the proposed NIR face presentation attack database.

**Ethnicity**	**Type**	**Sessions**	**Subjects**	**Videos**	**Images**	**Faces**
Indian	Real	1	152	152	36,480	32,629
Chinese	Real^*^	4	725	–	12,469	12,469

* *From NIR-VIS 2.0 database (Li et al., 2013)*.

### 2.3. Chinese Face Presentation Attack Database

To prepare the attack database with Chinese ethnicities, real visible spectrum frontal images of 98 subjects are randomly selected from the NIR-VIS 2.0 database (Li et al., 2013). Similar to the Indian database, these images are also printed on A4 paper using HP color printer. These prints are then placed on a fixed medium to capture the attack videos. A total of 98 videos comprising 7, 840 images form the NIR attacked subset while real NIR samples from 725 individuals acquired from the CASIA NIR-VIS2.0 database (Li et al., 2007) comprise the bonafide Chinese subset. In total, Chinese database contains 12, 469 real face images and 7, 799 attack face images. Characteristics of the database are given in Table 1 and sample images are shown in Figure 1B.

### 2.4. Experimental Protocol

To facilitate benchmarking the performance of different algorithms on this database, we propose a protocol as summarized in Table 2.

**Table 2:** Protocol for the proposed Spoof-in-NIR database experiments.

**Ethnicity**	**Session**		**Folds**	**Results Reported**
	**Real**	**Attack**		
Indian	1	1	15	Video and Frame
	1	2	15	Video and Frame
Chinese	1	1	5	Frame

Indian NIR dataset is divided into subject independent 15 folds, where at a time one fold is used for training the linear SVM classifier for attack detection, and the remaining folds are used for evaluating the classifier. In the Indian subset, attack videos are captured in two different sessions; therefore, the results for both the sessions are calculated separately. To report the results of a particular session, attack videos captured in that session are used. Similarly, the Chinese NIR database is divided into five random folds. Due to the unavailability of the videos in the real subset of the Chinese NIR dataset, only frame based results are calculated. For the Indian NIR database, both video based and frame based protocols are designed. In video-based protocol, every video is classified as real or attack, and in the frame-based protocol, an individual entity of a video (frame) is classified as real or attack. The protocol will help evaluate the algorithm trained in limited and constrained data settings while testing large-scale data coming from multiple folds. In place of single fold train-test evaluation, multi-folds ensure the extensive assessment and generalizability of the algorithm.

### 2.5. Vulnerability of Face Recognition Against Attack

Researchers have demonstrated that several publicly available commercial face recognition systems are prone to presentation attacks (Wen et al., 2015; Agarwal et al., 2017). To show the effect of the proposed NIR face database on the Commercial-Off-the-Shelf (COTS) systems, a face identification experiment is performed. The gallery images are single frontal images of each subject. For instance, the gallery for the Indian NIR attack database comprises 150 images, one for each subject. Each attack video is used as a probe to perform the identification using FaceVACS^3^. It is important to observe that with all the attacked images as a probe, COTS yields the rank-1 identification accuracy of 100% for the Chinese NIR subset while Indian NIR attack videos show more than 98% identification accuracy at rank-1. The face identification experiment thus shows the vulnerability of the existing face recognition system against the attack in the NIR spectrum.

## 3. Proposed Presentation Attack Detection Algorithm

**Figure 2:**
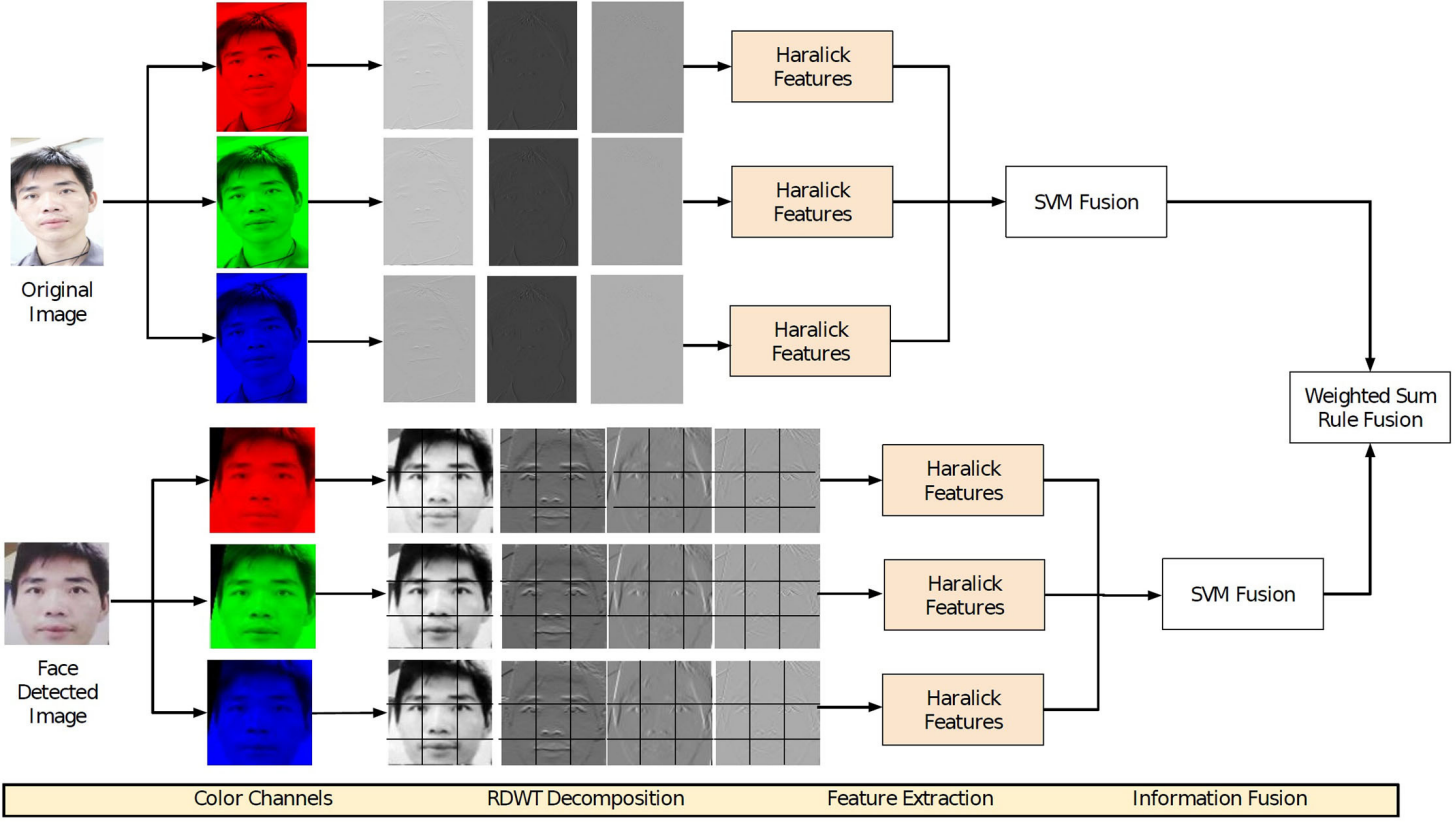
Illustrating the proposed presentation attack detection pipeline.

Face presentation attack detection algorithms proposed in the literature are primarily based on encoding texture measures and image artifacts such as moiŕe pattern (Patel et al., 2016), local binary patterns (Määttä et al., 2011; Boulkenafet et al., 2016; Ramachandra and Busch, 2017), and more recently, deep learning algorithms have been proposed (Chen et al., 2019; Tu et al., 2019; Ma et al., 2020). A recent study (Jia et al., 2020b) shows that hand-crafted image feature-based algorithms have the potential of handling multiple challenging presentation attacks while being computationally feasible. Therefore, in this article, we present a presentation attack detection algorithm based on combining the image features extracted from global as well as local facial regions. Raw images provide a global view of the real and attack data. This global information can help in extracting discriminative information such as foreground-background inconsistency and attacking medium boundary, while local facial regions may help in extracting textural information/artifact of the face. We hypothesize that since the attack images are mostly recaptured from the camera, they can be different in high-frequency content from its counterpart i.e., real face. The proposed presentation attack detection algorithm encodes these assertions to differentiate between real and attacked images. Figure 2 illustrates the steps involved in the proposed algorithm. To highlight the low and high frequency of discrimination, the first step of the algorithm involves applying the wavelet decomposition to the input image/frame. Wavelet provides the multi-resolution decomposition of the frame and highlights the edges in multiple directions: horizontal, vertical, and diagonal (Fowler, 2005). Discrete wavelet transform (DWT) downsamples the subbands to *M*/2 × *N*/2, where *M* and *N* are the height and width of the image respectively. Since downsampling leads to loss of information, we apply redundant discrete wavelet transform (RDWT). RDWT preserves the image size by creating subbands of the same size as the input image, thereby generating overcomplete representations.

As mentioned earlier, the effect of presentation attack may be visible in either facial regions or even as abnormalities concerning foreground and background regions. Therefore, wavelet decomposition is applied in two steps. In the first step, RDWT decomposition is applied on the complete input image without face detection, while the second step involves first applying face detection followed by tessellating the facial region into nine non-overlapping facial patches.

Global information, which is directly computed from the raw images, is computed without tessellating the frames into multiple blocks. To compute the information from the local texture, the face region is first divided into multiple blocks, and then each block is decomposed using a wavelet filter. The patches generated are of size 32 × 32. The textural features are then extracted by applying Haralick features (Haralick and Shanmugam, 1973) on the RDWT decomposed subbands, which can encode the image distortion-based information present in the recaptured images such as intensity distribution and homogeneity.

The basic building block of Haralick features is the Co-occurrence Matrix, which contains the information of counts of how many times a certain pixel value is in the neighborhood of other possible pixel values. The Haralick features used in this research are listed below:


$$\begin{array}{l} l_{i,j}=C_{i,j}/S \end{array}$$

where *C* is the Co-occurrence Matrix, and *S* is the sum of all elements of *C*. *l*_*i, j*_ is the likelihood of occurrence of the pixel value *i* in the neighborhood of pixel value *j*. *N*_*g*_ is the number of gray levels in matrix *C*.

- Global Homogeneity:

(1) $$\begin{array}{r} h_{1}=\overset{N_{g}}{\underset{r=1}{\sum }}\overset{N_{g}}{\underset{c=1}{\sum }}l{\left(r,c\right)}^{2} \end{array}$$

- Local Homogeneity:

(2) $$\begin{array}{r} h_{2}=\overset{N_{g}}{\underset{r=1}{\sum }}\overset{N_{g}}{\underset{c=1}{\sum }}\frac{1}{1+{\left(r-c\right)}^{2}}l\left(r,c\right) \end{array}$$

- Correlation Measures:

(3) $$\begin{array}{r} h_{3}=\frac{ERC-ERC_{1}}{\max\left\{ER,EC\right\}} \end{array}$$

(4) $$\begin{array}{r} h_{4}={\left(1-\exp\left\{-2\left(ERC_{2}-ERC\right)\right\}\right)}^{\frac{1}{2}} \end{array}$$

where,


$$\text{ERC}=-\overset{N_{g}}{\underset{r=1}{\sum }}\overset{N_{g}}{\underset{c=1}{\sum }}l\left(r,c\right)\log\left[\begin{array}{c} l\left(r,c\right) \end{array}\right]$$

$${\text{ERC}}_{1}=-\overset{N_{g}}{\underset{r=1}{\sum }}\overset{N_{g}}{\underset{c=1}{\sum }}l\left(r,c\right)\log\left\{l_{r}\left(r\right)l_{c}\left(c\right)\right\}$$

$${\text{ERC}}_{2}=-\overset{N_{g}}{\underset{r=1}{\sum }}\overset{N_{g}}{\underset{c=1}{\sum }}l_{r}\left(r\right)l_{c}\left(c\right)\log\left\{l_{r}\left(r\right)l_{c}\left(c\right)\right\}$$

- Average:

(5) $$\begin{array}{r} h_{5}=\overset{2N_{g}}{\underset{r=2}{\sum }}rl_{r+c}\left(r\right) \end{array}$$

- Intensity Variation:

(6) $$\begin{array}{r} h_{6}=\overset{N_{g}-1}{\underset{n=0}{\sum }}n^{2}\left\{\begin{array}{c} \overset{N_{g}}{\underset{r=1}{\sum }}\overset{N_{g}}{\underset{c=1}{\sum }}l\left(r,c\right) \end{array}\right\},|r-c|=n \end{array}$$

- Pixel Dependency:

(7) $$\begin{array}{r} h_{7}=\frac{\overset{N_{g}}{\underset{r=1}{\sum }}\overset{N_{g}}{\underset{c=1}{\sum }}\left(rc\right)l\left(r,c\right)-\mu_{r}\mu_{c}}{\sigma_{r}\sigma_{c}} \end{array}$$

- Variance

(8) $$\begin{array}{r} h_{8}=\overset{N_{g}}{\underset{r=1}{\sum }}\overset{N_{g}}{\underset{c=1}{\sum }}{\left(r-\mu \right)}^{2}l\left(r,c\right) \end{array}$$

- Total Variance:

(9) $$\begin{array}{r} h_{9}=\overset{2N_{g}}{\underset{r=2}{\sum }}{\left(r-h_{10}\right)}^{2}l_{r+c}\left(r\right) \end{array}$$

- Total Entropy:

(10) $$\begin{array}{r} h_{10}=-\overset{2N_{g}}{\underset{r=2}{\sum }}l_{r+c}\left(r\right)\log\left[\begin{array}{c} l_{r+c}\left(r\right) \end{array}\right] \end{array}$$

- Entropy:

(11) $$\begin{array}{r} h_{11}=-\overset{N_{g}}{\underset{r=1}{\sum }}\overset{N_{g}}{\underset{c=1}{\sum }}l\left(r,c\right)\log\left[\begin{array}{c} l\left(r,c\right) \end{array}\right] \end{array}$$

- Variance Difference:

(12) $$\begin{array}{r} h_{12}=\overset{N_{g}-1}{\underset{r=0}{\sum }}r^{2}l_{r-c}\left(r\right) \end{array}$$

- Entropy Difference:

(13) $$\begin{array}{r} h_{13}=-\overset{N_{g}-1}{\underset{r=0}{\sum }}l_{r-c}\left(r\right)\log\left[\begin{array}{c} l_{r-c}\left(r\right) \end{array}\right] \end{array}$$

Here, μ_*r*_, μ_*c*_, σ_*r*_, and σ_*c*_ are the mean and SDs of *l*_*r*_ and *l*_*c*_, respectively. *r* and *c* represent the row and column index in the co-occurrence matrix. *l*_*r*+*c*_(*r*) is the likelihood of co-occurrence matrix coordinates summing to *r*+*c*, and *ER*, and *EC* are the entropies of *l*_*r*_ and *l*_*c*_.

The process described above is repeated for each channel in the RGB image. While the Haralick features are used to encode the textural information, RDWT provides the high-frequency information presented in multiple orientations. For classification, a linear Support Vector Machine (SVM) (Vapnik, 2013) classifier is used based on its popularity in the PAD research (Ramachandra and Busch, 2017; Jia et al., 2020a). Two different SVM classifiers are trained: one for the features extracted from the raw sensor images and the second for the features extracted from the face region. The final score of a testing frame is calculated as the weighted sum of the scores obtained from two different trained SVM. To summarize, the steps involved in the proposed algorithm are discussed below:

1. The input image is decomposed into individual RGB channels
2. Each channel is then decomposed into four wavelet sub-bands using Redundant Discrete Wavelet Transform (RDWT) (Fowler, 2005),
3. Haralick texture features are computed over each wavelet sub-bands and the original image without decomposition,
4. Haralick features obtained from each subband are concatenated and input to a linear Support Vector Machine (SVM) classifier for classification,
5. The face region is cropped using the eye coordinates obtained using the Viola-Jones face detector,
6. Face region is divided into nine non-overlapping blocks,
7. Each face block is decomposed into four wavelet sub-bands using Redundant Discrete Wavelet Transform,
8. Haralick texture features are computed over each wavelet sub-bands and the original face block without decomposition,
9. Haralick features obtained from each subband and original face are concatenated and input to a linear Support Vector Machine (SVM) classifier for classification,
10. The scores obtained from SVM in Steps 4 and 9 are fused using weighted sum rule fusion and then thresholded for classification.

## 4. Results on Multispectral PAD Databases

The performance of the proposed algorithm is computed on the proposed Spoof-in-NIR database along with the publicly available multispectral MSSPOOF database (Chingovska et al., 2016) and the CASIA-SURF database (Zhang et al., 2020). The results are reported in terms of Bonafide (real) Presentation Classification Error Rate (BPCER), Attack Presentation Classification Error Rate (APCER), and Average Classification Error Rate (ACER) also known as Half Total Error Rate (HTER). BPCER is defined as the rate of the bonafide (real) samples classified as attack samples. APCER is the proportion of the attack samples classified as bonafide (real) samples. The results are also reported using Equal Error Rate (EER).


$$\text{APCER}=1-\frac{\overset{A}{\underset{i=1}{\sum }}\xi }{\left|A\right|}$$

$$\text{BPCER}=\frac{\overset{B}{\underset{i=1}{\sum }}\varsigma }{\left|B\right|}$$

ACER=(APCER+BPCER)/2

where, |*A*| and |*B*| is the total number of presentation attack and real images, respectively. ξ and ς is 0 if an attack image is classified as real (bonafide), else 1.


$$\text{FAR}=\frac{FalsePositive}{\left|A\right|}$$

$$\text{FRR}=\frac{FalseNegative}{\left|B\right|}$$

HTER=(FAR+FRR)/2

HTER is an average of FRR and FAR. EER is a specific value of HTER at which FAR is equal to FRR.

First, the results of the proposed algorithm on existing MSSPOOF and CASIA-SURF databases along with comparisons to state-of-the-art (SOTA) algorithms are reported. Later, the experiments performed on the proposed Spoof-in-NIR database are described. The comparison of the proposed algorithm with SOTA algorithms highlights the efficacy of PAD in multiple spectrums.

### 4.1. Results on MSSPOOF Database

Multispectral spoof database contains images with print attack performed in four different ways: 1) original image is captured in the visible spectrum, while the visible image is recaptured using visible and near infrared spectrum camera, and 2) original image is captured in near-infrared spectrum while NIR image is recaptured back using visible and near infrared filter camera. The database contains a total of 630 real images in visible and 624 images in near infrared spectrums pertaining to 21 subjects, in 7 different environmental conditions. The database is divided into three disjoint sets: train, dev, and test. Raghavendra et al. (2016) proposed two different protocols on the MSSPOOF database:

1. *Individual spectrum*: NIR and VIS, individual spectrum data in training and testing.
2. *Combined spectrum*: both spectrum data in training and testing.

We have reported the results using these two protocols along with the experiments defined in this paper to fully utilize the characteristics of the database. In the individual spectrum experiments: the presentation attack detection SVM model is trained using individual spectrum data such as for visible spectrum experiments. The partition belonging to the visible spectrum is used for training and evaluating the classifier. In the combined spectrum experiment, the train set belonging to both the spectra is used to learn the classifier, and similarly, for evaluation, test sets belonging to both spectra are combined.

#### 4.1.1. Results of Individual Spectrum Experiments

The results for individual spectrum experiments are summarized in Table 3. The proposed algorithm achieves 0% ACER and BPCER in both the spectrums which shows the consistency of the algorithm across spectrums. The second best performing algorithm is by Raghavendra et al. (2016) (L_*a*_MT_*i*_F) and it achieves 0.74% and 2.08% ACER in NIR and VIS spectrum, respectively. L_*a*_MT_*i*_F uses a combination of Laplacian pyramid based decomposition to extract the high-frequency information and Short Term Fourier Transform (STFT) for time and frequency features. The limitation of the L_*a*_MT_*i*_F algorithm is the high error rate for attack detection. Table 3 also shows the results of several other texture based algorithms and it can be observed that the algorithms are generally ineffective in detecting either the bonafide presentation or attack presentation. The proposed algorithm is robust to both kinds of data, which is desired and required for real-world presentation attack detection algorithms integrated with face recognition systems. The misclassification of bonafide data as attack data can frustrate the genuine user because of the need to give face data again and again for recognition. At the same time allowing attack data as bonafide data can cause serious harm to the face recognition system. The proposed algorithm yields the best EER (i.e., 0%) on the MSSPOOF database.

**Table 3:** Results (%) on individual and combined spectrum set of MSSPOOF database.

**Spectrum**	**VIS**			**NIR**			**VIS + NIR**		
**Algorithm**	**APCER**	**BPCER**	**ACER**	**APCER**	**BPCER**	**ACER**	**APCER**	**BPCER**	**ACER**
LBP-SVM^*^	2.31	11.67	6.99	0.46	8.33	4.39	2.54	7.77	5.16
BSIF-SVM^*^	5.55	4.44	5.00	4.16	2.22	3.19	3.47	3.33	3.40
LPQ-SVM^*^	5.55	0.55	3.05	0.92	4.44	2.68	1.85	4.44	3.14
DoG-SVM^*^	62.03	28.88	45.46	37.03	38.54	37.79	43.05	43.61	43.33
GLCM-SVM^*^	**0**	97.22	48.61	**0**	96.08	48.04	**0**	98.05	49.02
L_*a*_MT_*i*_F	4.16	**0**	**2.08**	0.92	**0.55**	**0.74**	3.00	**2.50**	**2.75**
Proposed	**0**	**0**	**0**	**0**	**0**	**0**	**0**	**0**	**0**

* *Results are taken from Raghavendra et al. (2016). The top two values are bolded*.

#### 4.1.2. Results on Combined Spectrum Experiments

In the combined spectrum experiment, the data belonging to both spectrums are utilized to make a joint decision. To learn the presentation attack detection model, the training set given in the database for both the spectrums is used and evaluation is performed on the test set of both the spectrums collectively. Table 3 shows the results of the proposed and existing algorithms on the combined set.

The proposed algorithm achieves 0.0% ACER on the combined spectrum database which is significantly better than existing algorithms. The results show that the proposed algorithm is robust towards different kinds of data. Similar to individual spectrum results, the combined spectrum shows the ineffectiveness of the existing algorithms in detecting attack or bonafide data. The GLCM features yield the perfect error rate in detecting the presentation attack but rejecting the bonafide almost all the time. Similarly, the second best algorithm yields 3% APCER. The proposed algorithm yields 1.39% EER on the joint spectrum dataset.

### 4.2. Experiments on CASIA-SURF Database

The recently proposed CASIA-SURF database (Zhang et al., 2020) is one of the most extensive databases for face presentation attack detection problems both in terms of modalities and subjects. The database consists of 21, 000 videos of 1,000 subjects in RGB (color), IR, and depth modalities. For each subject, one real video is captured while six fake videos are captured using eye, mouth, and nose regions cut from the flat and curved printed face. For example, in attacks 1 and 2, the eye region is cut from the flat and curved face photo. Similarly, in other attacks, either the eyes and nose or eyes, nose, and mouth, all portion is cut from flat and curved face photo. The color, depth, and IR videos are acquired using the Intel RealSense SR300 camera. The real faces are first printed out using an A4 color printer and later used by the attackers while exhibiting real life motions such as turning left or right, moving up or down, and walk-in or away from the camera. The database is divided into training, validation, and testing set and contains 148,089, 48,789, and 295,644 cropped images, respectively.

In this research, we have used the pre-defined protocol for evaluation, and the results are reported on fused modalities. The baseline algorithm (Zhang et al., 2019) consists of the ResNet-18 model as a backbone model where the first three ResNet blocks are used for feature extraction. The features from each modality, i.e., color, depth, and IR, are then fused using a squeeze and excitation module. In the end, two blocks of ResNet are used for discriminative features learning, followed by global average pooling. The whole pipeline is trained using a softmax classifier. The comparison of the proposed algorithm with the baseline algorithm in terms of error rates is given in Table 4.

**Table 4:** Error rates (%) of the proposed and baseline algorithms on the CASIA-SURF database (Zhang et al., 2019).

**Type**	**Algorithm**	**Modality**	**EER**	**APCER**	**BPCER**	**ACER**
Fused	Baseline	Color&IR	8.5	14.4	1.6	8.0
	Proposed		10.0	10.1	9.9	10.0
	Baseline	Color&Depth	6.2	4.3	5.6	5.0
	Proposed		1.8	1.7	1.9	1.8
	Baseline	Depth&IR	5.3	1.5	8.4	4.9
	Proposed		2.0	1.9	2.1	2.0
	Baseline	Color&Depth&IR	2.9	3.8	**1.0**	2.4
	Proposed		1.5	**1.6**	1.4	**1.5**

*Best result is bolded*.

**Figure 3:**
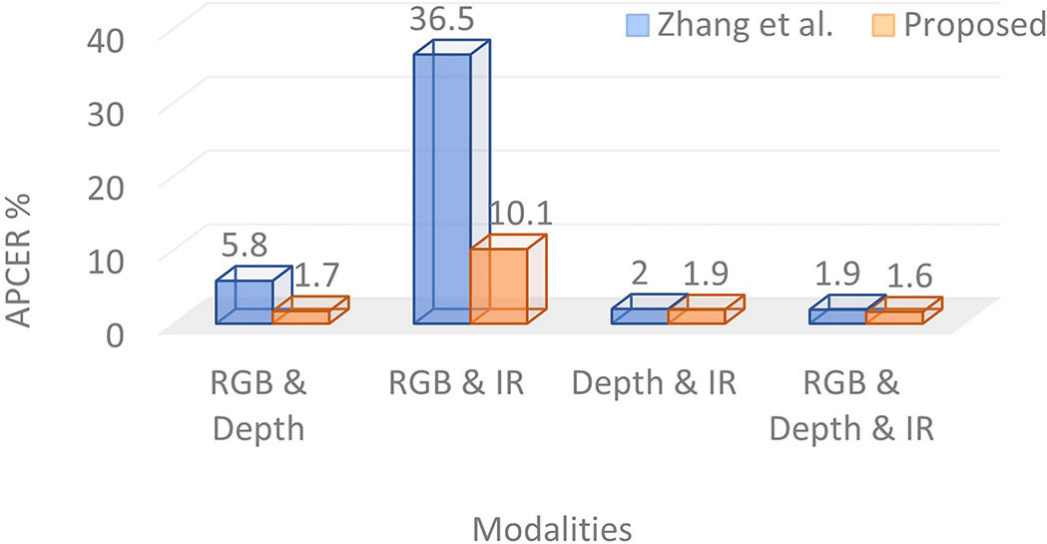
Comparing Attack Presentation Classification Error Rate (APCER) (%) of the proposed algorithm with Zhang et al. (2020).

The scores computed over different modalities are fused using a weighted sum. The weight parameter across different modalities is learned, which yields the lowest EER on the validation set. The fusion of all three modalities, i.e., color, depth, and IR, outperforms the baseline algorithm by 37.5% (i.e., reduces from 2.4% to 1.5%) in terms of ACER. The APCER of the baseline algorithm (i.e., 3.8%) is more than two times that of the proposed algorithm (i.e., 1.6%). The proposed fusion of color and IR with depth modality surpasses the baseline algorithm in identifying bonafide (i.e., real) images by at least 66%. As shown in Figure 3, the APCER of the proposed algorithm is significantly better than the recently proposed face PAD algorithm by Zhang et al. (2020). For example, when the fusion of color and IR data is performed, the APCER of the proposed and existing algorithm (Zhang et al., 2020) is 10.1% and 36.5%, respectively. The fusion of all modalities in the proposed algorithm yields 1.6% APCER, whereas, the existing algorithm yields 1.9% APCER. These superior performances of the proposed algorithm on one of the largest multi-modal presentation attack databases establish the efficacy of the proposed algorithm in identifying physical fake face data.

### 4.3. Experiments on Proposed Spoof-in-NIR Database

The proposed Spoof-in-NIR database contains images and videos of subjects from two different ethnicities: Indian and Chinese. Indian NIR database contains 152 real videos and 300 attack videos collected in two sessions. Chinese NIR database contains 12, 469 real frames taken from CASIA VIS-NIR 2.0 (Li et al., 2013) and 7, 799 attack frames. According to the protocol described in Section 2.4, the experiments are performed on the proposed Spoof-in-NIR database. To make our approach in-line with the recent literature (Zhang et al., 2019, 2020; Jia et al., 2020b) which has utilized the ResNet as a backbone or to make the comparison, we have also performed a comparison with the ResNet-18 model (He et al., 2016) pre-trained on ImageNet (Deng et al., 2009). The model is fine-tuned for PAD for 50 epochs using the Adam optimizer and adaptive learning rate, where the initial value is set to 0.0001.

The results of the proposed algorithm on the Spoof-in-NIR database are summarized in Table 5. The proposed algorithm achieves 4.3% and 0.8% EER on sessions 1 and 2 of the Indian NIR spectrum dataset, respectively, for video-based experiments. Similarly, for the frame-based evaluation, an EER of 19.7% and 23.2% is achieved for sessions 1 and 2, respectively. On the Chinese NIR dataset, the proposed algorithm yields 20.7% EER using a frame-based experiment. The reported results show that the classification of an individual frame is difficult compared to video, where the information from multiple frames helps improve the results. Apart from that, the proposed algorithm surpasses the deep CNN architecture, i.e., ResNet, by a significant margin. Due to the imbalanced nature of images/videos of real and spoof classes in the proposed dataset especially in the Chinese subset, we have also used another evaluation metric namely the weighted F-1 score. The weighted F-1 score on the Chinese PAD subset of the proposed and ResNet-18 architecture is 0.8230 and 0.6907, respectively. On the Indian subset, the average weighted F-1 values for video-based classification are 0.9687 and 0.8675 which are obtained from the proposed and ResNet algorithm, respectively. Comparing the performance with MSSPOOF (Chingovska et al., 2016) and CASIA-SURF (Zhang et al., 2020) databases further show the proposed database is more challenging. The availability of this database to the research community will further improve the state-of-the-art presentation attack detection in multiple spectrums.

**Table 5:** Video and frame based EER (μ±σ)% on the proposed NIR print attack database.

**Ethnicity**	**Session**	**Algorithm**	**Video**	**Frame**
Indian	1	ResNet-18	12.8 ± 3.5	24.1 ± 5.8
		Proposed	**4.3** **±2.3**	**19.7** **±1.9**
	2	ResNet-18	10.2 ± 0.9	29.3 ± 2.3
		Proposed	**0.8** **±0.6**	**23.2** **±1.6**
Chinese	1	ResNet-18	-	27.4 ± 3.0
		Proposed	-	**20.7** **±0.8**

*Top results are bolded*.

## 5. Experiments on Existing VIS Spectrum Databases

To further demonstrate the effectiveness of the second contribution of this article i.e., the development of a robust algorithm across different attacks, additional experiments are performed on the existing benchmark databases in the VIS spectrum. The performance of the proposed algorithm is evaluated on the CASIA-Face Anti-Spoofing Database (FASD) (Zhang et al., 2012), Replay-Attack (Chingovska et al., 2012), MSU-MFSD (Wen et al., 2015), 3D Mask Attack Database (3DMAD) (Erdogmus and Marcel, 2013), MSU USSA database (Patel et al., 2016), WMCA (George et al., 2019), SiW-M (Liu et al., 2019b), Silicone Mask Attack Database (SMAD) (Manjani et al., 2017), and 3D wax figure face database (WFFD) (Jia et al., 2019). These databases cover a wide spectrum of attacks such as print, photo, a replay of video, 3D hard resin masks, and the most challenging silicone mask. Table 6 summarizes the characteristics of these databases and a brief description of each is provided below.

**Table 6:** Characteristics of the existing VIS spectrum attack database used in this research.

**Database**	**Attack**	**Unconstrained**
CASIA-FASD	Print and Replay	✓
Replay-Attack	Print and Replay	✓
MSU-MFSD	Print and Replay	✓
3DMAD	3D Hard Resin Mask	×
MSU USSA	Print and Replay	✓
SMAD	Silicone Mask	✓
WFFD	3D Wax Figure	✓
WMCA	Print, Replay, and Mask	✓
SiW-M	Print, Replay, and Mask	✓

CASIA-FASD database (Zhang et al., 2012) contains three different kinds of attacks: cut photo (eye portions are cut to perform the eye blink), warped photo (to make it cylindrical as a real face), and replay of a video. It contains the videos in three different image qualities: low, normal, and high. Replay-Attack database (Chingovska et al., 2012) is captured in controlled and adverse environments. In the controlled environment, the background was kept fixed and the fluorescent lamp was used for illumination. In the adverse environment, the background is random and natural light is the source of illumination. MSU-MFSD database (Wen et al., 2015) is captured from 35 subjects and is one of the mobile face attack databases. Real videos are captured from two different devices: a built-in camera of a MacBook Air 13-inch laptop and a front facing camera of a Google Nexus 5 Android phone. To capture the attack, two different high-resolution cameras are used: a Canon 550D single-lens reflex camera and an iPhone 5S back facing camera.

CASIA-FASD, Replay-Attack, and MSU-MFSD databases are challenging but contain 2D attacks only. To assess the effectiveness of the algorithm on the 3D attack, the 3DMAD database (Erdogmus and Marcel, 2013) is also used in this article. Advancement in the 3D reconstruction and 3D printer makes the availability of 3D masks easier. These masks can be worn and can effectively hide the identity of the person in day-to-day life. These masks are hard to detect in comparison to wearing photo paper masks. 3DMAD database is captured from 17 subjects where each subject is wearing a different 3D mask. It is captured in three sessions, where the real access videos are captured in the first two sessions and the third session covers the 3D mask attack. For each subject ten real and five 3D attack videos are captured, with a total of 255 videos in the database. While the 3DMAD database is the challenging 3D mask attack database but it has some limitations. The masks used to prepare the database are hard resin masks that do not allow movements similar to the natural face. In the real world, some challenging cases are found where the robbers have used silicone masks to hide their identity from the surveillance cameras. These silicone masks are soft masks that can properly fit the face and can move with the face. Manjani et al. (2017) have prepared the Silicone Mask Attack Database (SMAD) which is currently the most challenging kind of attack to detect.

To tackle the limitations such as diversity in terms of background, illumination, and image quality, Patel et al. (2016) prepared one of the largest databases. Such a database is essential to obtain generalizable and robust anti-spoofing methods, particularly in face unlock scenarios on smartphones. To create such a database we selected 1,000 live subject images of celebrities from the Weakly Labeled Face Database^4^. The public set of the MSU USSA database for face anti-spoofing consists of 9, 360 images (out of which 1, 040 are real images and 8, 320 spoof attack images) of 1, 040 subjects. To perform the experiments standard database protocol of 5-fold cross-validation is performed.

On the grandtest protocol of the WMCA database, the proposed algorithm achieves 2.4% ACER; whereas, the existing algorithms such as FASNet (Lucena et al., 2017), DeepPixBis (George and Marcel, 2019), and MC-ResNetPAD (Parkin and Grinchuk, 2019) achieve 11.44%, 6.0%, and 2.6%, respectively. On the unseen protocols of SiW-M, the proposed algorithm achieves the average EER (%) and ACER (%) of 12.8% and 14.6%, respectively. The ACER (%) of Auxiliary (Liu et al., 2018), Deep Tree Network (Liu et al., 2019b), DeepPixBis (George and Marcel, 2019), and MCCNN (George and Marcel, 2020) is 23.6%, 16.8%, 19.6%, and 18.6%, respectively. The extensive results on these challenging databases further prove the effectiveness of the proposed algorithm.

Recently, a new modality of 3D attack is highlighted where wax figure faces are used as a possible adversary on face recognition systems (Jia et al., 2019). The authors have shown that state-of-the-art face recognition algorithms such as OpenFace (Amos et al., 2016) and Face++^5^ are vulnerable to wax figure faces. These spoof faces have achieved at least 92% Impostor Attack Presentation Match Rate (IAMPR) across multiple protocols. Therefore, the identification of wax faces from real faces is important and challenging because of properties similar to real faces. In this research, we have used two working conditions (protocols) provided by the authors. In the first protocol (Prot. 1), images captured under different recording devices and environments are used, whereas, in another protocol (Prot. 3), images captured in different and same recording devices and environments are combined. Protocol 1 consists of 600 trains, 200 development, and 440 test images; while, protocol 3 consists of 1,320 trains, 440 development, and 440 test images.

**Figure 4:**
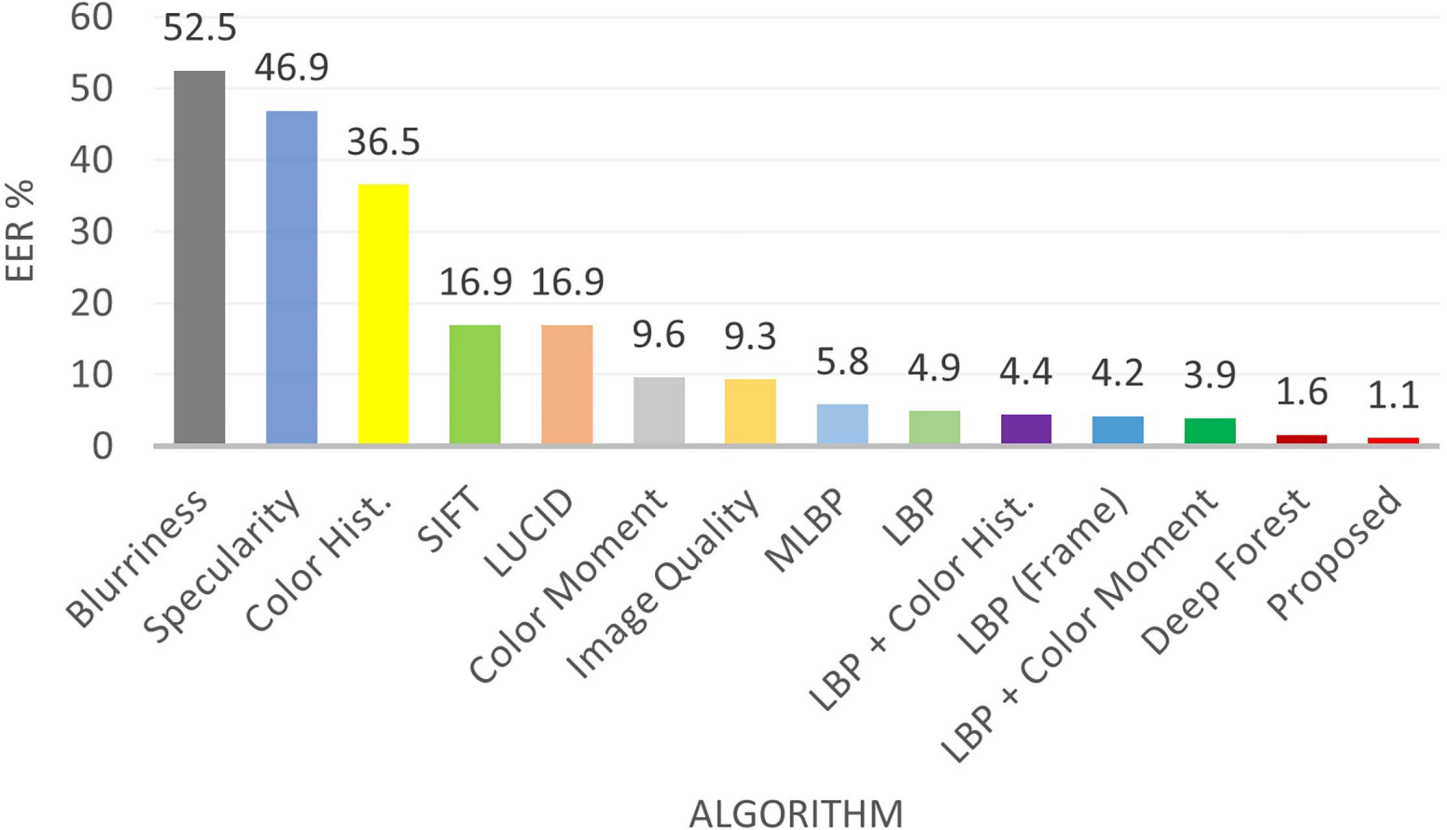
Comparison with existing results including deep forest (Cai and Chen, 2019) on the MSU USSA database for presentation attack detection.

To compare the results with existing state-of-the-art algorithms, the original protocol of each database is followed and the results are reported both in terms of intra-database and cross database scenarios. Table 7 shows the comparison of the proposed algorithm with existing algorithms in video based attack detection. On one of the most challenging presentation attack i.e., silicone mask, the proposed algorithm outperforms the state-of-the-art performances (Manjani et al., 2017; Shao et al., 2019). EER and HTER of the proposed algorithm on video-based detection are 7.7% and 6.9% which is more than 37% and 47% lower, respectively. Similarly, on the CASIA-FASD database, the proposed algorithm gives the lowest EER value of 0.92%. Perfect EER on Replay-Attack, MSU-MFSD, and 3DMAD shows the robustness of the algorithm across different attacks and acquisition/attack devices. The proposed algorithm outperforms various deep learning algorithms (Manjani et al., 2017; Tu and Fang, 2017; Ma et al., 2020; Pinto et al., 2020; Song et al., 2020), multi cue fusion algorithms (Patel et al., 2016; Zhang et al., 2018), motion algorithms (Edmunds and Caplier, 2018), and texture algorithms (Boulkenafet et al., 2016; Peng et al., 2020). The ultra deep neural network proposed by Tu and Fang (Tu and Fang, 2017) combines a pre-trained deep residual network with Long Short Term Memory (LSTM) and yields an EER of 1.22% and 1.03% on CASIA-FASD and Replay-Attack databases, respectively. The EER of the proposed algorithm is at least 24% better than (Tu and Fang, 2017) on CASIA-FASD while 0% EER is achieved on the Replay-Attack database. As shown in Figure 4, the proposed algorithm outperforms several state-of-the-art presentation attack detection algorithms including the recent deep forest (Cai and Chen, 2019) algorithm on one of the largest MSU-USSA databases. The EER and standard deviation of the proposed, Deep Forest (Cai and Chen, 2019) and LBP + Color moment (Patel et al., 2016) algorithm are 1.1±0.3%, 1.6±0.6%, and 3.9±0.8%, respectively.

**Table 7:** Comparison with existing results on the video based presentation attack detection.

**Algorithm**	**CASIA-FASD**	**Replay-Attack**		**MSU-MFSD**	**3DMAD**	**SMAD**	
	**EER**	**EER**	**HTER**	**EER**	**EER**	**EER**	**HTER**
Spectral Cubes (Pinto et al., 2015a)	14.0	–	2.8	–	–	–	–
DMD + LBP + SVM (Tirunagari et al., 2015)	21.8	5.3	3.8	–	–	–	–
Multicue Fusion (Patel et al., 2016)	5.88	–	14.6	8.41	–	–	–
Color Texture (Boulkenafet et al., 2016)	3.2	**0.0**	3.5	3.5	–	–	–
C-SURF + Fisher Vector (Boulkenafet et al., 2017a)	2.8	0.1	2.2	2.2	–	–	–
Deep Dictionary (Manjani et al., 2017)	**1.3**	–	**0.0**	–	**0.0**	**12.3**	13.1
LGBP + GS-LBP (Peng et al., 2017)	2.53	–	3.13	8.54	–	–	–
Directional LBP (Qin et al., 2017)	4.44	–	4.88	3.33	–	–	–
Frame Diff + Fisher Score + LPQ (Azeddine et al., 2017)	4.62	5.60	4.80	2.50	–	–	–
Depth and patch CNNs (Atoum et al., 2017)	2.67	0.79	0.72	–	–	–	–
Skin Blood Flow (Wang et al., 2017)	7.01	–	4.92	7.23	–	–	–
Multiscale quality (Yeh and Chang, 2018)	12.7	–	5.38	–	–	–	–
Temporal Texture (Pan and Deravi, 2018)	6.71	–	**0.6**	10.07	–	–	–
Motion CodeBook (Edmunds and Caplier, 2018)	17.0	–	5.7	17.0	3.53	–	–
Texture Markov Feature (Zhang et al., 2018)	8.0	4.0	4.4	7.5	–	–	–
3D CNN (Li et al., 2018)	1.4	0.3	1.2	**0.0**	–	–	–
Locally Specialized CNN (Gustavo et al., 2018)	4.44	0.33	1.75	–	–	–	–
CNN + STN+ MIL (Lin et al., 2018)	–	–	1.8	–	–	–	–
Deep Dynamic Texture (Shao et al., 2019)	–	–	–	–	**0.0**	14.9	**11.7**
GFA-CNN (Tu et al., 2019)	–	–	–	7.5	–	–	–
Spoof Buster (Bresan et al., 2019)	–	–	5.50	–	–	–	–
2-stream ResNet-18 + Attention (Chen et al., 2019)	3.15	0.21	0.39	–	–	–	–
Patch and Depth CNN-v2 (Liu et al., 2019a)	4.4	**0.0**	**0.0**	–	–	–	–
Multi-Regional CNN (Ma et al., 2020)	–	–	1.6	–	–	–	–
CCoLBP+Ensemble Learning (Peng et al., 2020)	3.33	–	4.00	5.00	–	–	–
Color Texture Weighted Features (Song et al., 2020)	7.34	2.32	7.39	–	–	–	–
SFDSF^*^ (Song et al., 2020)	15.38	5.15	6.06	–	–	–	–
FDCNN-AUTO^**^ (Song et al., 2020)	5.06	0.93	2.77	–	–	–	–
SfSNet (Pinto et al., 2020)	3.3	–	3.1	–	–	–	–
SE-ResNet18 (Wang et al., 2020)	3.3	–	1.3	6.3	–	–	–
**Proposed**	**0.92**	**0.0**	0.75	**0.0**	**0.0**	**7.7**	**6.9**

*Two best results are bolded*.

* *Spatial-Frequency Domain Selection Feature*

** *Features on Double Convolutional Neural Network and Autoencoder*.

Table 8 shows that the proposed algorithm either achieves state-of-the-art or competitive results even for frame-based classification with all the challenging face spoofing databases. The EER value of 0.8%, 4.95%, and 0.0% is achieved on Replay-Attack, CASIA-FASD, and MSU-MFSD database respectively in frame based detection. HTER of 2.1% is achieved on the Replay-Attack database in the grand test attack scenario which is lower than various texture based algorithms (Boulkenafet et al., 2016; Peng et al., 2017). On the SMAD database, the proposed algorithm shows an improvement of more than 25% to 47% from the baseline performance (Manjani et al., 2017). The detection error rate using the proposed and existing algorithms on wax figure faces is reported in Table 9. Similar to other challenging attacks, the proposed algorithm outperforms several existing algorithms for wax face detection including hand-crafted and deep learning algorithms. The ACER of the proposed algorithm is 17.02% better than VGG-16 based wax face detection.

**Table 8:** Comparison with SOTA results on the frame based presentation attack detection in terms of EEE (%) and HTER (%).

**Algorithm**	**CASIA-FASD**	**Replay-Attack**		**MSU-MFSD**	**SMAD**	
	**EER**	**EER**	**HTER**	**EER**	**EER**	**HTER**
Motion (Anjos and Marcel, 2011)	26.6	11.6	11.7	–	–	–
LBP (Chingovska et al., 2012)	18.2	13.9	13.8	–	–	–
CDD (Yang et al., 2013)	11.8	–	–	–	–	–
Motion + LBP (Komulainen et al., 2013)	–	4.5	5.1	–	–	–
LBP-TOP (de Freitas Pereira et al., 2014)	–	7.9	7.6	–	–	–
IQA (Galbally et al., 2014)	32.4	–	15.2	–	–	–
CNN (Yang et al., 2014)	7.4	6.1	**2.1**	–	–	–
IDA (Wen et al., 2015)	–	–	7.4	8.5	–	–
Color Texture (Boulkenafet et al., 2016)	**2.1**	**0.4**	2.8	**4.9**	–	–
LGBP + GS-LBP (Peng et al., 2017)	**2.5**	–	3.13	8.54	–	–
Deep Dictionary (Manjani et al., 2017)	–	–	–	–	**14.7**	**15.0**
**Proposed**	4.95	**0.8**	**2.1**	**0.0**	**10.9**	**10.7**

*Two best results are bolded*.

**Table 9:** Wax figure face detection error rates (%) on the unconstrained (protocol 1) and real-world protocol (protocol 3) of WFFD database (Jia et al., 2019).

**Algorithm**	**EER**			**APCER**			**BPCER**			**ACER**		
	**Prot. 1**	**Prot. 3**	**Avg**.	**Prot. 1**	**Prot. 3**	**Avg**.	**Prot. 1**	**Prot. 3**	**Avg**.	**Prot. 1**	**Prot. 3**	**Avg**.
M-Scale LBP	33.17	34.56	**33.86**	31.22	33.33	**32.27**	31.22	32.92	**32.07**	31.22	33.13	**32.17**
Color LBP	33.17	36.81	34.99	30.24	35.38	32.81	36.10	35.79	35.94	33.17	35.58	34.37
Reflectance	41.95	44.78	43.36	40.00	46.01	43.00	52.19	46.22	49.20	46.10	46.11	46.10
VGG-16	45.85	48.67	47.26	50.73	45.19	47.96	41.95	49.28	45.61	46.34	47.24	46.79
Proposed	23.50	35.68	**29.59**	25.50	35.68	**30.59**	22.00	35.91	**28.95**	23.75	35.79	**29.77**

*The proposed algorithm reduces the average classification error rate (ACER) and EER by 2.40% and 4.27%, respectively. Two Best results are bolded*.

The proposed algorithm utilizes the weighted score fusion of the classifier trained using the features computed by tessellating the face region and full image input. Therefore, to study the importance of the individual region and segregate them into small regions, we have performed experiments on multiple databases. Interestingly, it is found that the features from the global region without input tessellation are useful in handling presentation attacks. For example, on MSU-MFSD and Replay-Attack, the raw input images show effectiveness in detecting the presentation attacks by achieving at most 0.4% EER. The fusion of face and full image further improves the detection performance. On CASIA-FASD, the fusion reduces the EER from 1.1% (face region) to 0.92% (face + raw image).

To further show the generalizability of the proposed PAD algorithm, cross database experiments are also performed, and the results are reported in Table 10. For video based scenarios, when the anti-spoofing algorithm is trained on the CASIA-FASD database, average HTER values of 26.7% and 35.3% are reported on the MSU-MFSD and Replay-Attack databases, respectively. The average HTER on CASIA-FASD and Replay-Attack is 23.7% and 32.2% respectively when the anti-spoofing model is trained using the MSU-MFSD database. When the model is learned using the Replay-Attack database and tested on each subset of CASIA-FASD and MSU-MFSD the average HTER value reported is 33.3% and 23.9%, respectively. The anti-spoof model trained using the Replay-Attack database shows better generalizability and may be attributed to the fact that it is captured in different illumination, devices, and background. The comparison of the proposed countermeasure with the existing anti-spoofing algorithms is shown in Table 10.

**Table 10:** Comparison with existing results on the video based presentation attack detection under cross dataset setting.

**Train database**	**Algorithm**	**Test database**		
		**CASIA-FASD**	**MSU-MFSD**	**Replay-attack**
CASIA-FASD (Zhang et al., 2012)	Motion (de Freitas Pereira et al., 2013)	–	–	50.2
	Spectral Cubes (Pinto et al., 2015b)	–	–	34.4
	LBP (Boulkenafet et al., 2015)	–	36.6	47.0
	Color Texture (Boulkenafet et al., 2016)	–	20.4	30.3
	LBP+ GS-LBP (Peng et al., 2017)	–	**18.6**	48.4
	Directional LBP (Qin et al., 2017)	–	26.3	21.6
	Frame Diff + Multi-Level + Fisher Score + LPQ (Azeddine et al., 2017)	–	50.4	50.3
	Multiscale quality (Yeh and Chang, 2018)	–	–	38.1
	De-Spoofing (Jourabloo et al., 2018)	–	–	28.5
	Texture Markov Feature (Zhang et al., 2018)	–	32.4	32.3
	Motion CodeBook (Edmunds and Caplier, 2018)	–	50.0	33.7
	Spoof Buster (Bresan et al., 2019)	–	–	53.0
	Two stream ResNet-18 + Attention (Chen et al., 2019)	–	–	36.2
	Patch and Depth CNN-v2 w/o update (Liu et al., 2019a)	–	–	34.7
	Patch and Depth CNN-v2 (Liu et al., 2019a)	–	–	**15.4**
	CCoLBP+Ensemble Learning (Peng et al., 2020)	–	**18.6**	**18.7**
	SAPLC (Sun et al., 2020a)	–	–	27.3
	FCN-LSA (Sun et al., 2020b)	–	–	27.3
	**Proposed**	–	26.7	35.3
Replay-Attack (Chingovska et al., 2012)	Motion (de Freitas Pereira et al., 2013)	47.9	–	–
	Spectral Cubes (Pinto et al., 2015b)	50.0	–	–
	LBP (Boulkenafet et al., 2015)	39.6	35.2	–
	Color Texture (Boulkenafet et al., 2016)	37.7	34.1	–
	LBP+ GS-LBP (Peng et al., 2017)	40.3	36.1	–
	Directional LBP (Qin et al., 2017)	46.6	31.1	–
	Frame Diff + Multi-Level + Fisher Score + LPQ (Azeddine et al., 2017)	42.6	38.0	–
	Multiscale quality (Yeh and Chang, 2018)	39.0	–	–
	De-Spoofing (Jourabloo et al., 2018)	41.1	–	–
	Texture Markov Feature (Zhang et al., 2018)	45.9	37.7	–
	Motion CodeBook (Edmunds and Caplier, 2018)	49.3	40.8	–
	Spoof Buster (Bresan et al., 2019)	43.3	–	–
	Two stream ResNet-18 + Attention (Chen et al., 2019)	34.7	–	–
	Patch and Depth CNN-v2 w/o update (Liu et al., 2019a)	36.1	–	–
	Patch and Depth CNN-v2 (Liu et al., 2019a)	**23.2**	–	–
	CCoLBP+Ensemble Learning (Peng et al., 2020)	39.3	**25.0**	–
	SAPLC (Sun et al., 2020a)	37.5	–	–
	FCN-LSA (Sun et al., 2020b)	37.3	–	–
	**Proposed**	**33.3**	**23.9**	–
MSU-MFSD (Wen et al., 2015)	LBP (Boulkenafet et al., 2015)	49.6	–	42.0
	Color Texture (Boulkenafet et al., 2016)	46.0	–	33.9
	LBP+ GS-LBP (Peng et al., 2017)	40.6	–	45.3
	Directional LBP (Qin et al., 2017)	40.2	–	48.8
	Frame Diff + Multi-Level + Fisher Score + LPQ (Azeddine et al., 2017)	50.0	–	48.0
	Texture Markov Feature (Zhang et al., 2018)	57.0	–	42.7
	Motion CodeBook (Edmunds and Caplier, 2018)	47.7	–	30.6
	CCoLBP+Ensemble Learning (Peng et al., 2020)	**39.6**	–	**27.2**
	**Proposed**	**23.7**	–	**32.3**

*The results are reported in terms of average HTER (%). HTER of the cross database experiments and comparison with State-of-the-art results using video based countermeasure. Results of Spoof Buster (Bresan et al., 2019) are reported when a single database is used in training. (Top two results are bolded)*.

The proposed algorithm outperforms the state-of-the-results when the countermeasure is trained using Replay-Attack and MSU-MFSD database. The proposed algorithm improves the HTER of the 2nd best performing algorithm (Peng et al., 2020) from 39.6% to 23.7% on the CASIA-FASD database when the model is trained on MSU-MFSD. Similarly, the proposed algorithm improves the performance on MSU-MFSD by 1.1% when the classifier is trained using Replay-Attack. Recently proposed algorithms based on deep CNN by Chen et al. (2019) and Sun et al. (2020b) yield an HTER value of 34.7% and 37.3% on the CASIA database when the model is trained on Replay-Attack, whereas the HTER of the proposed algorithm is at least 1.4% lower. The patch and depth CNN-v2 (Liu et al., 2019a) outperform the proposed algorithm in a cross-database setting; however, the significant drawbacks of the algorithm are the processing speed and high memory requirement. As claimed by the authors, their algorithm can process 1–2 frames per second, hence challenging for large scale implementation or deployment on mobile devices. Simultaneously, the proposed algorithm is highly computationally inexpensive both in terms of speed and memory requirement. Another strength of the proposed algorithm concerning patch and depth CNN-v2 (Liu et al., 2019a) is that it outperforms under the same database but unseen subjects train-test setting (reported in Table 7).

**Figure 5:**
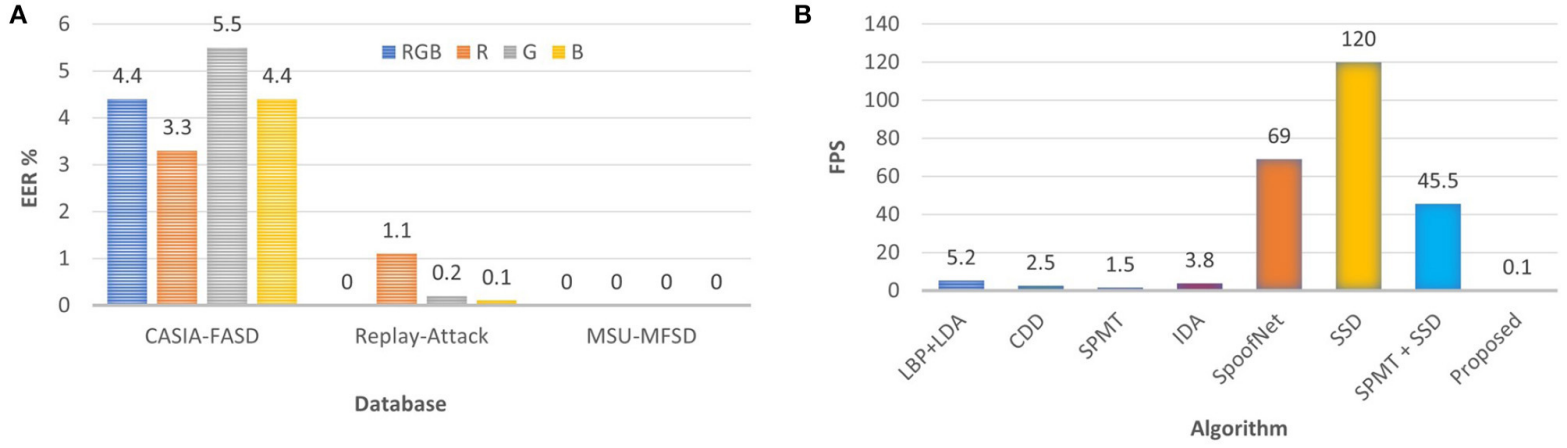
Ablation study of the proposed PAD algorithm in terms of its performance on the individual color channel of the images. Apart from that the practicality of the proposed algorithm to be deployed in resource constraint devices using computational speed. **(A)** Classification results with individual color channel and RGB; **(B)** Computational complexity of the PAD algorithms.

We have also performed the presentation attack detection using the individual color channel of RGB. To perform these experiments, random 30 frames from each video are first selected for feature extraction. The performance is reported on three challenging benchmark databases: CASIA-FASD, Replay-Attack, and MSU-MFSD. Results with 30 frames of a train and test video on the CASIA-FASD, Replay-Attack, and MSU-MFSD databases are reported in Figure 5A. R channel yields the lowest EER value of 3.3% on CASIA-FASD database which is lower than (Feng et al., 2016; Patel et al., 2016). On the Replay-Attack database, the lowest EER value of 0.1% is given by the B channel which is equal to the EER reported by Boulkenafet et al. (2017a). Boulkenafet et al. (2017a) have used all frames of a video while we have used only 30 frames for such comparable performance.

**Efficacy of the proposed algorithm:** In summary, the strengths of the proposed generalized PAD algorithm are listed below:

- The proposed algorithm can be implemented in real time. The feature extraction time on core i7@ 3.4GHz CPU machine with a Matlab environment is 0.1 frames per second (FPS) (Figure 5B). The huge deployment of face unlocking on mobile devices^6^ needs protection from presentation attacks. The proposed algorithm with such low computational time and memory requirement can also be implemented on mobile devices;
- The proposed algorithm outperforms various state-of-the-algorithms including 3D CNN (Li et al., 2018), SfSNet (Pinto et al., 2020), Multi-Regional CNN (Ma et al., 2020), deep dictionary (Manjani et al., 2017), and SE-ResNet18 (Wang et al., 2020), for a variety of presentation attacks including silicone mask attack (Manjani et al., 2017) and 2D attacks;
- The proposed algorithm is also able to handle new modality of 3D attacks i.e., wax faces (Jia et al., 2019). The average EER of the proposed algorithm is at least 37.4% and 12.6% lower than VGG-16 deep learning and multi-scale (M-scale) LBP texture features;
- The proposed algorithm is generalizable across imaging spectrum (VIS/NIR), attacks (2D/3D), acquisition devices (Mobile/High-def), and quality of images (Low/High).

## 6. Conclusion

Similar to the visible spectrum, face recognition in the near-infrared (NIR) spectrum is also vulnerable to presentation attacks. In the literature, there is very limited research on developing efficient and inclusive countermeasures for the attack in the NIR spectrum and designing a unified algorithm to design and evaluate the performance of PAD algorithms toward continuously evolving presentation attacks in multiple spectra. In this research, we contribute to this space by creating a large NIR PAD face database that comprises videos with different kinds of attacks on Indian and Chinese ethnicities. We next present a presentation attack detection algorithm for efficiently differentiating between bonafide and attacked images in the NIR spectrum. The generalizability of the proposed algorithm is demonstrated by evaluating the performance of 11 existing databases and comparing it with state-of-the-art results reported in the literature. It is observed that the proposed algorithm yields the best results on almost all the databases using all three metrics of APCER, BPCER, and EER. In cross-database evaluations, while the proposed algorithm yields the best results, the error rates are comparatively higher. In future study, we plan to improve the effectiveness of the algorithm so that the error rates can be further reduced without increasing the computational complexity.

## Data Availability Statement

The raw data supporting the conclusions of this article will be made available by the authors, without undue reservation.

## Ethics Statement

Written informed consent was obtained from the individual(s) for the publication of any potentially identifiable images or data included in this article.

## Author Contributions

AA conducted the experiment(s) and analyzed the results. All authors conceived the proposed algorithm and experiment(s), reviewed and updated the article, and performed the proofreading of the paper.

## Funding

AA was partially supported by the Visvesvaraya Ph.D. Fellowship. MV is partially supported through the Swarnajayanti Fellowship by the Government of India. This research is partially supported through a research grant from the Ministry of Electronics and Information Technology, Government of India.

## Conflict of Interest

The authors declare that the research was conducted in the absence of any commercial or financial relationships that could be construed as a potential conflict of interest.

## Publisher's Note

All claims expressed in this article are solely those of the authors and do not necessarily represent those of their affiliated organizations, or those of the publisher, the editors and the reviewers. Any product that may be evaluated in this article, or claim that may be made by its manufacturer, is not guaranteed or endorsed by the publisher.

## Footnotes

^1^ http://iab-rubric.org/resources.html

^2^ http://www.jai.com/en/products/go-5000-usb

^3^ http://www.cognitec.com

^4^ http://wlfdb.stevenhoi.com

^5^ https://www.faceplusplus.com/face-compare-sdk/

^6^ www.counterpointresearch.com/one-billion-smartphones-feature-face-recognition-2020/
